# High-Urgency Renal Transplantation: Indications and Long-Term Outcomes

**DOI:** 10.1155/2013/314239

**Published:** 2013-02-07

**Authors:** Lampros Kousoulas, Nikos Emmanouilidis, Wilfried Gwinner, Jürgen Klempnauer, Frank Lehner

**Affiliations:** ^1^Department of General, Visceral and Transplant Surgery, Hannover Medical School, 30625 Hanover, Germany; ^2^Department of Nephrology and Hypertension, Hannover Medical School, 30625 Hanover, Germany

## Abstract

The concept of high-urgency (HU) renal transplantation was introduced in order to offer to patients, who are not able to undergo long-term dialysis treatment, a suitable renal graft in a short period of time, overcoming by this way the obstacle of the prolonged time spent on the waiting list. The goal of this study was to evaluate the patient and graft survivals after HU renal transplantation and compare them to the long-term outcomes of the non-high-urgency renal transplant recipients. The clinical course of 33 HU renal transplant recipients operated on at our center between 1995 and 2010 was retrospectively analyzed. The major indication for the HU renal transplantation was the imminent lack of access for either hemodialysis or peritoneal dialysis (67%). The patient survival of the study population was 67%, 56%, and 56%, whereas the graft survival was 47%, 35% and 35%, at 5, 10, and 15 years, respectively. In the comparison between our study population and the non-HU renal transplant recipients, our study population presented statistically significant (*P* < 0.05) lower patient survival rates. The HU renal transplant recipients also presented lower graft survival rates, but statistical significance (*P* < 0.05) was reached only in the 5-year graft survival rate.

## 1. Introduction

Renal transplantation is the treatment of choice for patients with end-stage renal disease, as it increases the survival of the recipients and improves their quality of life, as compared to long-term dialysis treatment [1–3]. As the number of patients in need of renal transplantation rapidly increases, whereas the supply of organs available for transplantation stays stable or even decreases in some countries [4], the prolonged time spent on the waiting list for transplantation is nowadays a cardinal problem for the majority of patients and especially for those who are not able to undergo dialysis treatment or for those who develop severe complications of the end-stage renal disease [5–7]. To overcome this obstacle, the concept of high-urgency (HU) renal transplantation was introduced by Eurotransplant, in order to offer to this group of patients a renal graft in a short period of time. The major indications for a HU renal transplantation are the imminent lack of access for either hemodialysis or peritoneal dialysis and the inability of the patient to cope with dialysis with a high risk for suicide. Moreover, severe uremic polyneuropathy and severe bladder problems (such as hematuria and cystitis) due to kidney graft failure after a combined kidney-pancreas transplantation are also indications for a HU renal transplantation [8]. Although the option of high-urgency renal transplantation exists almost from the beginning of transplantation, little is known on the long-term patient and graft survivals of this special group of renal transplant recipients. The primary results reported seem to be rather disappointing, with a graft survival of 59% and patient survival of 84% at two years respectively [9].

The goal of this study was to evaluate the patient and graft survivals after HU renal transplantation and compare them to the long-term outcomes of the non-high-urgency renal transplant recipients. 

## 2. Patients and Methods

From the total of the 2937 renal transplantations performed in the Hanover Medical School from January 1995 to December 2010, thirty-three were high-urgency transplantations (1.1%). The perioperative data of the high-urgency renal transplant recipients were retrospectively analysed, and the survival of the patients was checked with the German residence registration offices, the general practitioners of the recipients, and our interdisciplinary outpatient clinic for renal transplant recipients. Systematic follow up of all cases was carried out until 01.08.2012.

Recipient demographics, the etiology of renal insufficiency, and the indications for the HU renal transplantation are given in Table 1. 

The average age of the study population at the time point of renal transplantation was 38 years and ranged from 1 to 65 years of age. Four recipients (12%) were children. The gender distribution of the recipients was 48% females (*n* = 16) and 52% males (*n* = 17). The average time spent on the high-urgency waiting list was 71 days. In 55% of the cases the high-urgency transplantation was a retransplantation. All high-urgency renal transplantations were performed as cadaveric transplants, and no recipient was highly immunized (panel reactive antibodies more than 86%). The organ quality was rated as good in all of the transplantations. 

The major indication for the high-urgency renal transplantation was the lack of dialysis access in 67% of the cases (*n* = 22). Five patients had to be transplanted on the high-urgency status because of severe psychological problems, four patients because of severe complications of hemodialysis (severe hypotension during the hemodialysis treatment), and two because of uremic polyneuropathy. 

The patient and graft survivals of our study population (*n* = 33) were compared to the patient and graft survivals of the non-high-urgency patients (*n* = 2904) transplanted in our department between 1995 and 2010. 

There were no statistically significant differences observed between the high-urgency renal transplant recipients and the non-high-urgency recipients regarding demographics, etiology of renal insufficiency, the percentage of retransplantation, and the immunological status. 

The study was reviewed by the local ethic committee and was performed in accordance with the Declaration of Helsinki. 

## 3. Statistical Analysis

The follow up of the HU-renal transplant recipients until August 2012 was based on data collected in our interdisciplinary outpatient clinic for renal transplant recipients. The AMIS/Windows version 1.0 software package was used (Hanover Medical School, Hannover, Germany). For statistical analysis, the SPSS version 20.0 software program was used (SPSS Inc., Chicago, IL, USA). The Kaplan-Meier analysis was used for the study endpoints patient and graft survivals. The Mann-Whitney test was used for comparisons between the study population and the non-HU renal transplant recipients. 

## 4. Results 

An overview of all patients is given in Table 2. 

### 4.1. Patient Survival

**Figure 1:**
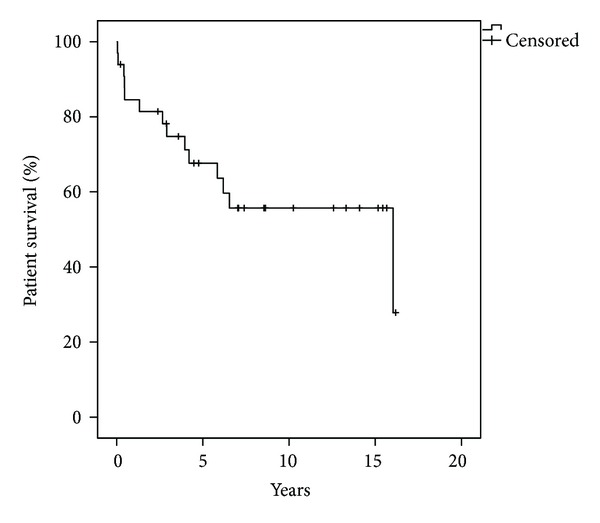
Patient survival after high-urgency renal transplantation.

From the thirty-three high-urgency renal transplant recipients, fourteen patients (42%) died. The average patient survival after the renal transplantation was 6.7 years. All of the patients died without a functioning graft. Out of these fourteen patients only one (patient # 29) died shortly after the renal transplantation (17 days) due to sepsis and multiple organ failure after the renal graft was lost because of a venous thrombosis. All of the other deaths were not associated with the renal transplantation. The major causes of death were sepsis and multiple organ failure in five patients, myocardial infarction in two patients, and subdural bleeding in two patients. The etiology of renal insufficiency and the indication for the high-urgency renal transplantation had no effect on the survival of the recipients. Moreover, nineteen of the HU renal transplant recipients are still alive, eleven of them still have a good renal function, but eight of them have lost the renal graft and have returned back to long-term dialysis treatment. The patient survival was 67%, 56% and 56% at 5, 10, and 15 years, respectively (Figure 1). In the comparison between our study population and the non-high-urgency renal transplant recipients our study population presented statistically significant (*P* < 0.05) lower survival rates (Table 3), but there was no difference observed regarding the cause of death of the recipients between these two groups. 

### 4.2. Graft Survival

**Figure 2:**
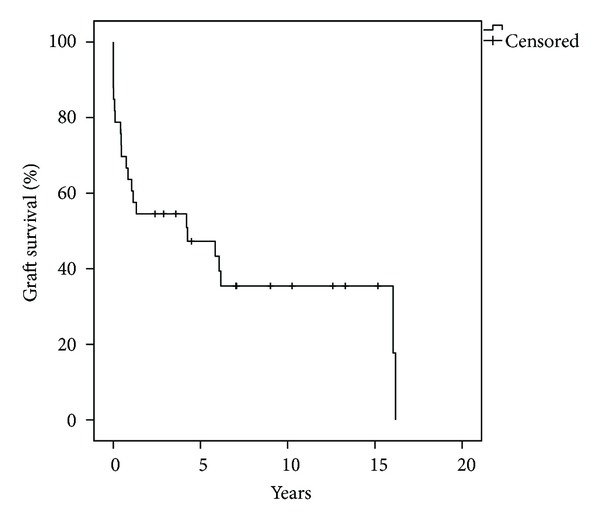
Graft survival after high-urgency renal transplantation.

From the total of the thirty-three patients who received a high-urgency renal transplantation, twenty-two (67%) lost the renal graft. The average graft survival was 4.6 years. In eight cases the cause of graft loss was the death of the patient; in all these cases the renal graft lost the function shortly before the death of the recipient because of multiple organ failure. In the rest of the cases (*n* = 14) the patients who lost the graft returned to long-term dialysis treatment, but six of them died in an average time of 2.5 years. The major reasons for graft loss were immunological complications in fives cases (three patients with chronic rejection and two patients with acute rejection), vascular complications in four cases (three patients with venous thrombosis and one patient with arterial thrombosis), initial graft failure in four cases (defined as the need for postoperative dialysis), and one patient lost the graft because of the development of glomerulonephritis. It is important to mention that our study population presented more immunological and vascular complications in comparison to the non-high-urgency renal transplant recipients. The etiology of renal insufficiency and the indication for the high-urgency renal transplantation had no effect on the graft survival. The 5-, 10-, and 15-year graft survival was 47%, 35%, and 35%, respectively (Figure 2). Our study population presented lower graft survival rates in comparison to the non-high-urgency renal graft recipients (*n* = 2904), but a statistical significance (*P* < 0.05) was reached only in the 5-year survival rate (Table 4). 

## 5. Discussion

The concept of high-urgency renal transplantation was introduced in order to offer rescue transplantation to a special group of patients who are not able to undergo long-term dialysis treatment. The aim of this study was to evaluate the long-term outcomes of the high-urgency renal transplant program of our department and compare them to the outcomes of the non-high-urgency renal transplantations. 

This single-center study presented patient survival rates of 67%, 56%, and 56% at 5, 10, and 15 years, respectively. It is important to mention that from the total of the fourteen patients who died after the high-urgency renal transplantation, only one death was related to the transplantation procedure, as the patient suffered a sepsis with multiple organ failure due to the lost of the renal graft because of thrombosis of the transplant renal vein. In the rest of the cases the death was not associated to the renal transplantation and the average patient survival after the transplantation was 6.7 years. Sepsis with multiple organ failure, myocardial infarction, and subdural bleeding were the main causes of death. Our findings correlate with the patient survival rates presented by De Meester et al. who demonstrated a two-year patient survival of 84% for the total of 161 high-urgency patients transplanted between 1993 and 1996 [9]. In the comparison of our study population to the non-high-urgency renal transplant recipients, the HU recipients presented statistically significant lower survival rates confirming the inferior outcomes after HU renal transplantation. In our opinion, the lower patient survival after HU renal transplantation is mainly attributable to the lower graft survival rates of patients after HU renal transplantation. As there was no difference observed regarding the cause of death of the recipients between the two groups and as only one death was related to the transplantation procedure, it has to be assumed that the early loss of the renal grafts in this group of patients has a negative effect on the survival of the patient. 

Regarding the graft survival of our study population, our study presented survival rates of 47%, 35%, and 35% at 5, 10, and 15 years, respectively. In eight cases the cause of graft loss was the death of the patient. In the rest of the cases (*n* = 14) the recipients lost the renal graft because of rejection, vascular complications, or because of primary nonfunction of the renal graft. Our data correlate with the numbers presented by De Meester et al who showed a poor outcome with a 2-year graft survival of 59% [9]. In the comparison between our study population and the non-high urgency renal transplant recipients, our patients presented lower graft survival rates, but a statistically significant difference was observed only in the 5-year graft survival (47% versus 70%). The lower graft survival rates, especially at five years after the transplantation, could be explained by the high incidence of immunological and vascular complications presented in the group of HU renal transplant recipients. More specifically almost 35% of the recipients lost the renal graft because of acute or chronic rejection, rate which is clearly higher compared to the non-HU renal transplant recipients. The high incidence of immunological complications can be explained by the fact that the HU renal transplantations were performed only in presence of a negative cross-match regardless of the HLA matching. The worst HLA matching of this group of patients is probably responsible for the high incidence of acute and chronic rejection observed. Moreover, almost 29% of the HU recipients lost the renal graft because of vascular complications. In the majority of these patients the indication for the HU renal transplantation was the lack of access for hemodialysis which in most cases reflects the poor vascular condition (e.g., atheromatosis) of the recipients or even some types of coagulopathy. This is a possible explanation, as 75% of the patients that lost the graft because of arterial or venous thrombosis were listed as HU recipients because of the lack of access for hemodialysis mainly due to shunt thrombosis. 

Our study has some potential limitations. First of all, wanting to assess only the high-urgency renal transplant recipients transplanted between 1995 and 2010 we had to focus on thirty-three patients and exclude the rest of the renal transplant recipients. This selection may be a source of bias resulting in an underestimation of the factors affecting the patient and graft survivals after HU renal transplantation. It also has to be mentioned that the statistical power of our analyses is limited as our sample size of thirty-three patients is relatively small. This could be a possible reason why no statistically significant difference was reached in the comparison of the graft survival or why the etiology of the renal insufficiency and the indication for the HU transplantation had no effect on patient and graft survivals. 

To conclude, our study demonstrated poor long-term outcomes after high-urgency renal transplantation, as the patient and graft survivals, especially in the early period after the transplantation, were statistically significantly lower compared to the results of the non-HU renal transplant recipients. Living in a period of shortage of organs, further studies are needed in order to evaluate the results after high-urgency renal transplantation and identify the patients who really benefit from an HU renal transplantation. 

## Figures and Tables

**Table 1:** Recipient demographics, etiology of the renal disease, and indication for HU renal transplantation.

Number of patients	33
Gender (male/female) (%)	17/16 (52/48)
Average age at time of transplantation (years)	38 (1–65)
Average time on HU status (days)	71 (0–967)
Etiology of renal insufficiency	
Reflux nephropathy	8
Glomerulonephritis	6
IgA nephropathy	4
Diabetic nephropathy	3
Polycystic kidney disease	3
Vascular nephropathy	3
Analgetic nephropathy	2
Other	4
Indication for high-urgency renal transplantation (%)	
Lack of dialysis access	22 (67%)
Psychological problems	5 (15%)
Hemodialysis problems	4 (12%)
Uremic polyneuropathy	2 (6%)

**Table 2:** Characteristics of the 33 high-urgency renal transplant recipients assessed in our study.

Patient no	Age at transplantation (years)	Gender	Time of HU status (days)	Diagnosis	Reason for HU	Graft loss	Cause of graft loss	Graft survival (years)	Recipient status	Patient survival (years)	Cause of death
1	59	m	14	Vascular nephropathy	Uremic polyneuropathy	Yes	Death	16,0	Dead	16,0	Rupture of aortic aneurysm
2	29	m	0	Hemolytic uremic syndrome	Lack of dialysis access	Yes	Chronic rejection	16,2	Living	16,2	—
3	37	f	47	Reflux nephropathy	Lack of dialysis access	Yes	Death	4,2	Dead	4,2	Myocardial infarction
4	29	m	2	Reflux nephropathy	Lack of dialysis access	Yes	Chronic rejection	0,8	Living	15,7	—
5	12	m	4	Analgetic nephropathy	Hemodialysis problems	Yes	Death	0,5	Dead	0,5	Subdural bleeding
6	37	f	10	Reflux nephropathy	Lack of dialysis access	Yes	Glomerulonephritis	6,1	Living	15,4	—
7	53	m	1	IgA nephropathy	Uremic polyneuropathy	No	—	15,2	Living	15,2	—
8	61	m	1	Vascular nephropathy	Lack of dialysis access	Yes	Death	0,4	Dead	0,4	Myocardial infarction
9	26	f	92	Glomerulonephritis	Lack of dialysis access	Yes	Venous thrombosis	0,0	Living	14,1	—
10	35	m	5	Glomerulonephritis	Psychological problems	No	—	13,2	Living	13,3	—
11	46	m	26	IgA nephropathy	Psychological problems	Yes	Initial graft failure	0,0	Dead	4,0	Renal cell carcinoma
12	47	m	10	Diabetic nephropathy	Psychological problems	Yes	Acute rejection	4,3	Living	4,8	—
13	45	f	46	Tubulointerstitial nephritis	Hemodialysis problems	No	—	12,6	Living	12,6	—
14	41	m	69	Diabetic nephropathy	Hemodialysis problems	Yes	Arterial thrombosis	0,7	Dead	6,5	Sepsis due to leg amputation
15	48	m	504	Glomerulonephritis	Lack of dialysis access	Yes	Initial graft failure	5,8	Dead	5,8	Intestinal ischemia
16	19	f	117	Reflux nephropathy	Lack of dialysis access	Yes	Initial graft failure	0,0	Dead	2,7	Catheter associated sepsis
17	59	m	5	IgA nephropathy	Lack of dialysis access	Yes	Death	1,3	Dead	1,3	Sepsis due to leg amputation
18	38	f	25	Polycystic Kidney disease	Psychological problems	No	—	10,2	Living	10,2	—
19	38	f	1	Glomerulonephritis	Lack of dialysis access	Yes	Death	0,1	Dead	0,1	Sepsis due to pancreatitis
20	49	m	3	Vascular nephropathy	Lack of dialysis access	No	—	8,6	Living	8,6	—
21	44	m	967	Diabetic nephropathy	Hemodialysis problems	Yes	Initial graft failure	1,1	Dead	2,9	Sepsis due to leg amputation
22	30	f	260	Reflux nephropathy	Lack of dialysis access	Yes	Acute rejection	0,0	Living	8,5	—
23	65	f	1	Tubulointerstitial nephritis	Lack of dialysis access	Yes	Death	6,2	Dead	6,2	Pneumonia
24	31	f	16	Reflux nephropathy	Lack of dialysis access	Yes	Acute rejection	1,1	Living	7,4	—
25	53	f	2	Glomerulonephritis	Lack of dialysis access	No	—	7,1	Living	7,1	—
26	22	f	5	Reflux nephropathy	Lack of dialysis access	No	—	7,0	Living	7,0	—
27	1	f	7	Polycystic kidney disease	Lack of dialysis access	Yes	Death	0,4	Dead	0,4	Subdural bleeding
28	56	f	10	Analgesic nephropathy	Lack of dialysis access	No	—	4,5	Living	4,5	—
29	49	f	1	Glomerulonephritis	Lack of dialysis access	Yes	Venous thrombosis	0,0	Dead	0,0	Sepsis due to transplantation
30	2	m	50	Reflux nephropathy	Lack of dialysis access	No	—	3,6	Living	3,6	—
31	35	m	29	IgA nephropathy	Lack of dialysis access	Yes	Arterial thrombosis	0,1	Living	0,2	—
32	9	m	11	Unknown	Psychological problems	No	—	2,9	Living	2,9	—
33	48	f	14	Polycystic kidney disease	Lack of dialysis access	No	—	2,4	Living	2,4	—

**Table 3:** Comparison of patient survival between HU and non-HU renal transplant recipients.

	*n*	5 years	10 years	15 years
High-urgency renal transplant recipients	33	67%	56%	56%
Non-high-urgency renal transplant recipients	2904	90%	82%	78%
*P* value		*P* < 0.05	*P* < 0.05	*P* < 0.05

NS: nonsignificant; HU: high urgency.

**Table 4:** Comparison of graft survival between HU and non-HU renal transplant recipients.

	*n*	5 years	10 years	15 years
High-urgency renal transplant recipients	33	47%	35%	35%
Non-high-urgency renal transplant recipients	2904	70%	50%	36%
*P* value		*P* < 0.05	NS	NS

NS: nonsignificant; HU: high urgency.
